# A systematic investigation of the protein kinases involved in NMDA receptor-dependent LTD: evidence for a role of GSK-3 but not other serine/threonine kinases

**DOI:** 10.1186/1756-6606-2-22

**Published:** 2009-07-07

**Authors:** Stéphane Peineau, Céline S Nicolas, Zuner A Bortolotto, Ratan V Bhat, W Jonathan Ryves, Adrian J Harwood, Pascal Dournaud, Stephen M Fitzjohn, Graham L Collingridge

**Affiliations:** 1 MRC Centre for Synaptic Plasticity, Department of Anatomy, School of Medical Sciences, University Walk, Bristol, BS8 1TD, UK; 2 INSERM, U676, Robert Debré Hospital, 48 bvd Sérurier, 75019 Paris, France; 3 AstraZeneca R&D, SE-151 85 Södertälje, Sweden; 4 Cardiff School of Biosciences, Museum Avenue, PO Box 911, CF10 3US Cardiff, UK

## Abstract

**Background:**

The signalling mechanisms involved in the induction of N-methyl-D-aspartate (NMDA) receptor-dependent long-term depression (LTD) in the hippocampus are poorly understood. Numerous studies have presented evidence both for and against a variety of second messengers systems being involved in LTD induction. Here we provide the first systematic investigation of the involvement of serine/threonine (ser/thr) protein kinases in NMDAR-LTD, using whole-cell recordings from CA1 pyramidal neurons.

**Results:**

Using a panel of 23 inhibitors individually loaded into the recorded neurons, we can discount the involvement of at least 57 kinases, including PKA, PKC, CaMKII, p38 MAPK and DYRK1A. However, we have been able to confirm a role for the ser/thr protein kinase, glycogen synthase kinase 3 (GSK-3).

**Conclusion:**

The present study is the first to investigate the role of 58 ser/thr protein kinases in LTD in the same study. Of these 58 protein kinases, we have found evidence for the involvement of only one, GSK-3, in LTD.

## Background

A primary function of synapses is to store information by alterations in their efficiency of transmission. There are two major forms of long-lasting synaptic plasticity, long-term potentiation (LTP) and LTD, and these have been best characterised at synapses in the hippocampus [1,2]. The most extensively studied forms of both LTP and LTD are triggered by the synaptic activation of one class of glutamate receptor, the NMDA receptor, and are expressed to a large extent as alterations in synaptic transmission mediated by another class of glutamate receptor, the α-amino-3-hydroxy-5-methyl-4-isoxazole propionic acid (AMPA) receptor [3-5]. With respect to NMDA receptor-dependent LTD (NMDAR-LTD) it is generally believed that the process is expressed by the internalisation of AMPARs from the plasma membrane, resulting in a reduction in the number of AMPARs at synapses [6,7]. However, how the transient activation of NMDARs leads to this process is not well understood.

The first step involves Ca^2+ ^entry via NMDARs [8] and Ca^2+ ^release from intracellular stores [9,10]. Several Ca^2+^-dependent proteins have then been implicated in the process, including calmodulin [11], hippocalcin [12] and protein interacting with C-kinase 1 (PICK1) [13]. There is also strong evidence for the involvement of a ser/thr protein phosphatases cascade involving protein phosphatase 2B (calcineurin) and protein phosphatase 1 [11,14]. In addition, there is also evidence for the involvement of various protein kinases in hippocampal NMDAR-LTD, including cAMP-dependent protein kinase (PKA) [15,16], cyclin-dependent kinase 5 (CDK5) [17], mitogen-activated protein kinase 14 (p38 MAPK) [18] and glycogen synthase kinase-3 β (GSK3-β) [19]. However, the role of protein kinases has often not been substantiated and is, in some cases, controversial. In addition, the role of many protein kinases in LTD has not yet been investigated.

In the present study we have examined the role of 58 protein kinases in hippocampal NMDAR-LTD in slices obtained from two-week old rats. Inhibitors were applied directly to the cell under investigation via the patch-pipette, to avoid potential problems of access and to minimise the possibility of presynaptic effects. Based on these experiments, we can discount an involvement of at least 57 ser/thr protein kinases, but we are able to confirm a role for GSK-3. Thus, LTD not only involves high affinity Ca^2+^-sensors and protein phosphatases but also a ser/thr kinase. A major challenge for the future will be to establish the interactions between these various proteins during LTD.

## Methods

Experiments were performed on 400 μm thick parasagittal hippocampal slices obtained from juvenile (13 – 17 day old) rats. Procedures involving animals and their care were conducted in conformity with the institutional guidelines that are in compliance with national (UK animals (Scientific Procedures) Act 1986 and D.L.n.116, G.U., Suppl. 40, 1992) and international laws and policies (EEC Council Directive 86/609, OJ L 358, 1, 12 December 1987; Guide for the Care and Use of Laboratory Animals, U.S. National Research Council, 1996).

The slices were perfused with artificial cerebrospinal fluid (ACSF) which comprised (mM): NaCl, 124; KCl, 3; NaHCO_3_, 26; NaH_2_PO_4_, 1.25; CaCl_2_, 2; MgSO_4_, 1; glucose, 15; ascorbate, 2; (-)-bicuculline methochloride, 0.01. Visually-guided, whole-cell recordings were obtained at room temperature from the soma of CA1 neurons using patch electrodes that contained (mM): CsMeSO_4_, 130; HEPES, 10; NaCl, 8; EGTA, 0.5; Mg-ATP, 4; Na-GTP, 0.3; QX-314, 5. Schaffer collateral-commissural fibres were stimulated at a frequency of 0.1 Hz and excitatory postsynaptic current (EPSC) amplitude and access resistance recorded on-line at a holding potential of -70 mV. To attempt to induce NMDAR-dependent LTD, we delivered 300 pulses (at 0.66 Hz) at -40 mV, 20 to 40 minutes after formation of the whole-cell configuration [19]. Under control conditions this usually induced a robust LTD. Provided LTD was induced in the controls, experiments were interleaved in which various kinase inhibitors were included in the patch solution. Data were stored and analysed using the LTP Program [20,21] and are presented as mean ± s.e.m.

The magnitude of LTD was determined by comparing the average amplitude of responses over a 5 min period obtained immediately before and at least 20 min following the LTD induction protocol. To compare the magnitude of LTD in the different conditions, a non-parametric one-way ANOVA was performed. Significance was set at P < 0.05.

The following compounds were included in the whole-cell solution: Akt-I-1/2 (Akt1/2 kinase inhibitor, 1,3-dihydro-1-(1-((4-(6-phenyl-1H-imidazo [4,5-g]quinoxalin-7-yl)phenyl)methyl)-4-piperidinyl)-2H-benzimidazol-2-one hydrate trifluoroacetate salt), DMSO (dimethyl sulfoxide), H-89 (N-[2-(p-bromocinnamylamino)ethyl]-5-isoquinolinesulfonamide dihydrochloride), (all from Sigma-Aldrich, St. Louis, MO), Bis-1 (bisindolylmaleimide I, 2-[1-(3-dimethylaminopropyl)-1H-indol-3-yl]-3-(1H-indol-3-yl)-maleimide), DMAT (2-dimethylamino-4,5,6,7-tetrabromo-1H-benzimidazole), EGCG (()-epigallocatechin gallate, (2R,3R)-2-(3,4,5-trihydroxyphenyl)-3,4-dihydro-1 [2H]-benzopyran-3,5,7-triol-3-(3,4,5-trihydroxybenzoate), H-8 (N-[2-(methylamino)ethyl]-5-isoquinolinesulfonamide, 2HCl), IC261 (3-[(2,4,6-trimethoxyphenyl)methylidenyl]-indolin-2-one), IP3K inhibitor (inositol-1,4,5-trisphosphate 3-kinase inhibitor, N2-(m-trifluorobenzyl), N6-(p-nitrobenzyl)purine), LY294002 (2-(4-morpholinyl)-8-phenyl-4H-1-benzopyran-4-one), KN62 (4-[(2S)-2-[(5-isoquinolinylsulfonyl)methylamino]-3-oxo-3-(4-phenyl-1-piperazinyl)propyl] phenyl isoquinolinesulfonic acid ester), KT5720 ((9R,10S,12S)-2,3,9,10,11,12-hexahydro-10-hydroxy-9-meth yl-1-oxo-9,12-epoxy-1H-diindolo [1,2,3-fg:3',2',1'-kl]pyrrolo [3,4-i][1,6]benzodiazocine-10-carboxylic acid, hexyl ester), SB203580 (4-[5-(4-fluorophenyl)-2-[4-(methylsulfonyl)phenyl]-1H-imidazol-4-yl]pyridine), SP600125 (anthra [1-9-cd]pyrazol-6(2H)-one), U0126 (1,4-diamino-2,3-dicyano-1,4-bis [2-aminophenylthio]butadiene) (all from Tocris Cookson, Avonmouth, UK), CT99021 (6-{2-[4-(2,4-dichloro-phenyl)-5-(4-methyl-1H-imidazol-2-yl)-pyrimidin-2-ylamino]-ethylamino}-nicotinonitrile), (provided by Prof. P. Cohen, University of Dundee, UK), AR-164 (3-amino-6-{3-fluoro-4-[(4-methylpiperazin-1-yl)sulfonyl]phenyl}-N-pyridin-3-ylpyrazine-2-carboxamide) (synthesised as described previously [22]), PenGSKi (a 26-mer phosphopeptide rqikiwfqnrrmkwkkpltapsps*lq (s* = Phosphoserine)) and PenCTRL (penetratin peptide rqikiwfqnrrmkwkk) (synthesized for Prof A.J. Harwood and W.J. Ryves by Zinsser Analytic, UK).

Appropriate stock solutions were made and diluted with intracellular solution just before use.

## Results

**Figure 1:**
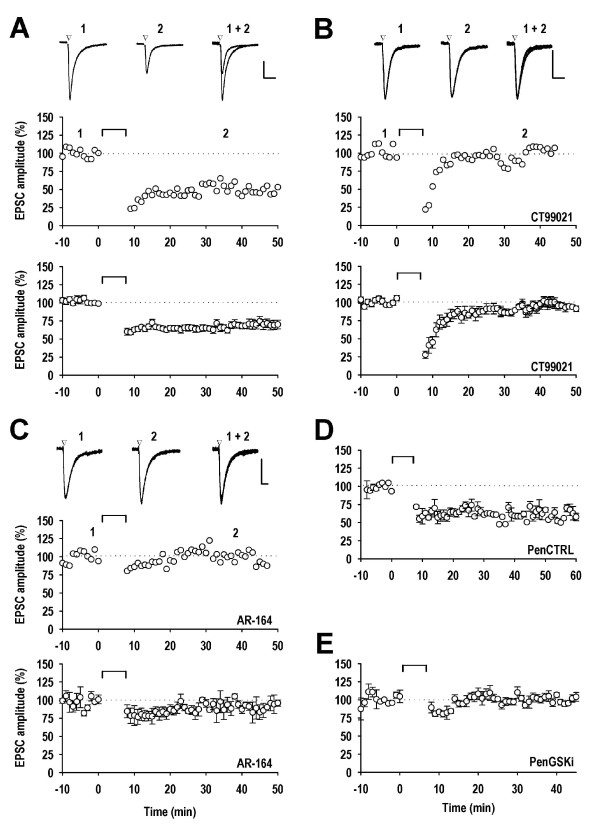
**GSK-3 inhibitors block the induction of LTD**. ***A***, A single experiment (upper) and pooled data from 28 experiments (lower) illustrating LTD under control conditions. ***B***, A single experiment (upper) and pooled data from 6 experiments (lower) illustrating the block of LTD by CT99021 (1 μM). ***C***, A single experiment (upper) and pooled data from 5 experiments (lower) illustrating the block of LTD by AR-164 (1 μM). ***D***, Pooled data from 3 experiments illustrating the effect of PenCTRL (20 μM) on LTD. ***E***, Pooled data from 3 experiments illustrating blockade of LTD by penGSKi (20 μM). In each panel, the points are the average amplitude of 6 successive EPSCs normalised with respect to the baseline. At t = 0, the neuron was depolarised to -40 mV and stimuli delivered at 0.66 Hz to the test input for the duration indicated by the bar. EPSCs (average of 6 consecutive records) obtained before and following the induction of LTD are illustrated at the times indicated (1, 2). The calibration bars for the traces depict 50 pA and 50 ms.

LTD was routinely induced in interleaved control neurons by delivering 300 pulses at -40 mV [23]. This resulted in a stable depression of the conditioned input, quantified 20 min following pairing, to 63 ± 2% of baseline (n = 28; Figure 1A). Inclusion of 0.5% DMSO, used as a solvent in some of the protein kinase experiments, had no effect on LTD (63 ± 3%; n = 7).

### Further Evidence for a role of GSK-3 in LTD

We previously proposed that activation of GSK-3 is required for LTD based on the sensitivity of this process to three structurally-unrelated inhibitors, SB415286, kenpaullone and lithium. However, none of these inhibitors are entirely specific for GSK-3 [24]. We therefore tested three additional inhibitors, which are believed to be more selective for GSK-3. First we examined CT99021 (1 μM), since this was recommended as the most selective GSK-3 inhibitor in a recent systematic analysis [24]. This compound invariably blocked the induction of LTD (98 ± 2%; n = 6; Figure 1B). The second GSK-3 inhibitor we examined, AR-164, also invariably blocked the induction of LTD (1 μM: 92 ± 3%; n = 5; Figure 1C; 5 μM: 97 ± 2%; n = 8; data not shown). Next we examined the effect of PenGSKi. This peptide features a cell-penetrating motif coupled to a GSK-3 inhibitor peptide and inhibits neuronal GSK-3 in vitro in a substrate-dependent manner with a Ki of 9 μM. This compound also blocked LTD whereas its control peptide did not (20 μM PenCTRL, 62 ± 3%; n = 3; Figure 1D and 20 μM PenGSKi, 96 ± 1%; n = 3; Figure 1E).

### Lack of evidence for a role of other ser/thr protein kinases in LTD

**Figure 2:**
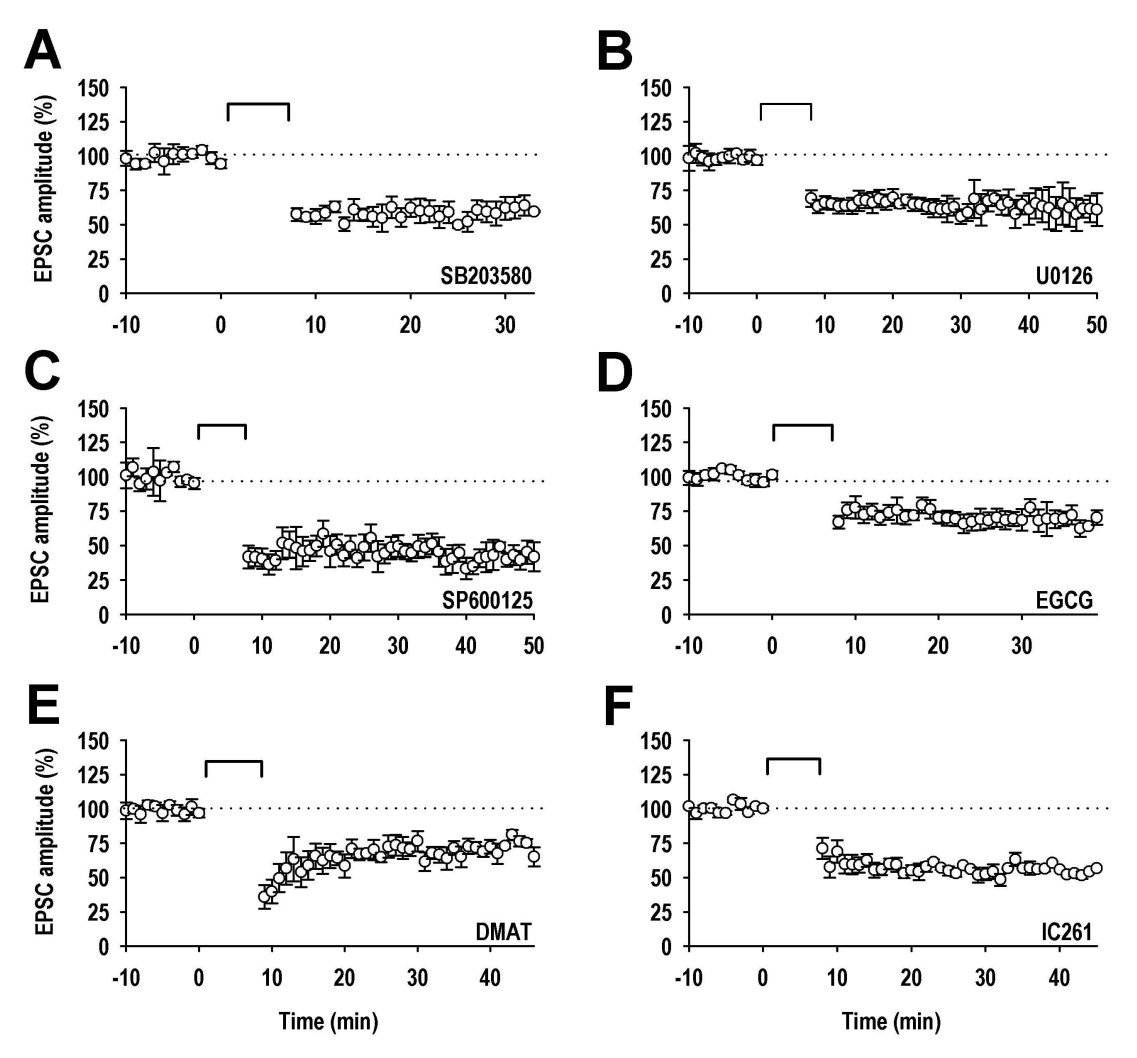
**Lack of effect of other CMGC group kinases inhibitors and a CK1 inhibitor on LTD**. ***A***, Pooled data (n = 7) illustrating the effects of SB203580 (5 μM). ***B***, Pooled data (n = 6) illustrating the effects of U0126 (20 μM). ***C***, Pooled data (n = 5) illustrating the effects of SP600125 (20 μM). ***D***, Pooled data (n = 6) illustrating the effects of EGCG (10 μM). ***E***, Pooled data (n = 5) illustrating the effects of DMAT (1 μM). ***F***, Pooled data (n = 6) illustrating the effects of IC261 (50 μM).

Whilst these data strongly implicate GSK-3 in LTD, they do not exclude a role for other ser/thr kinases, either operating in parallel with GSK-3 or acting in concert, perhaps as a priming kinase. We therefore systematically explored whether other ser/thr kinases were involved by testing a range of different inhibitors, selected for their known activity at the kinase under investigation. The protein kinases of the mammalian genome can be divided into several groups [25]. We started with the kinases that, like GSK-3, also belong to the CMGC group. Of these, the mitogen-activated protein kinases (MAPKs) are strongly implicated in various forms of synaptic plasticity [26]. However, neither the p38 MAPK inhibitor SB203580 (5 μM; 61 ± 5%; n = 7; Figure 2A), the mitogen-activated/extracellular signal regulated kinase (MEK) inhibitor U0126 (20 μM; 64 ± 4%; n = 6; Figure 2B) or the mitogen-activated protein kinase 8, 9 and 10 (JNK1, 2 and 3, respectively) inhibitor SP600125 (20 μM; 52 ± 5%; n = 5; Figure 2C) had any effect on LTD. We next tested inhibitors of the dual specificity tyrosine phosphorylation-regulated kinase (DYRK1A) and casein kinase 2 (CK2). Their respective inhibitors EGCG (10 μM) and DMAT (1 μM) were also without effect on LTD (70 ± 5%; n = 6, and 69 ± 6%; n = 5 respectively; Figure 2D and 2E). The potential role of casein kinase 1 (CK1), the prototypic member of the CK1 group of protein kinases, was tested using IC261 (50 μM); this inhibitor was also found to have no effect on LTD (60 ± 5%; n = 6; Figure 2F).

**Figure 3:**
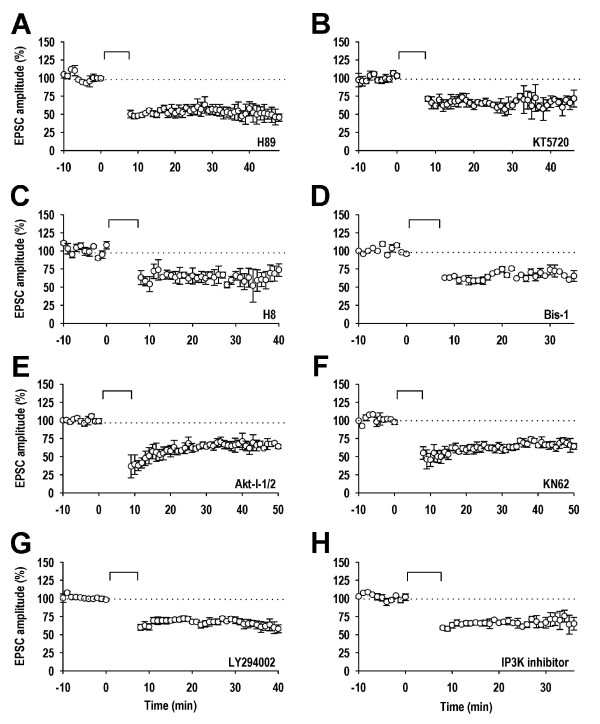
**Lack of effect of AGC, CAMK and lipid group kinase inhibitors on LTD**. ***A***, Pooled data (n = 5) illustrating the effects of H-89 (10 μM). ***B***, Pooled data (n = 4) illustrating the effects of KT5720 (1 μM). ***C***, Pooled data (n = 3) illustrating the effects of H-8 (10 μM). ***D***, Pooled data (n = 3) illustrating the effects of Bis-1 (1 μM). ***E***, Pooled data (n = 4) illustrating the effects of Akt-I-1/2 (10 μM). ***F***, Pooled data (n = 4) illustrating the effects of KN-62 (3 μM). ***G***, Pooled data (n = 5) illustrating the effects of LY294002 (10 μM). ***H***, Pooled data (n = 3) illustrating the effects of the IP3K inhibitor (20 μM).

The AGC group of protein kinases include several family members, such as protein kinase A (PKA), cyclic GMP-dependent protein kinase (PKG), and protein kinase C (PKC), that have been implicated in synaptic plasticity. However, in contrast to the GSK-3 inhibitors, PKA (10 μM H-89; 55 ± 3%; n = 5; Figure 3A or 1 μM KT5720; 68 ± 5%; n = 4; Figure 3B), PKG (10 μM H-8; 64 ± 6%; n = 3; Figure 3C) and PKC (1 μM Bis-1; 62 ± 6%; n = 3; Figure 3D) inhibitors had no effect on LTD. We previously reported that proto-oncogene proteins c-akt/protein kinase B (Akt/PKB), a downstream effector of phosphatidylinositol 3-kinase (PI3K), is not required for LTD, using a number of different strategies (blocking antibody, false substrate, dominant negative). Here we have extended this observation using a chemical inhibitor of this enzyme Akt-I-1/2 (10 μM; 67 ± 3%; n = 4; Figure 3E).

Calcium/calmodulin-dependent protein kinase II (CaMKII) is a member of the CAMK group of kinases and has been extensively studied in synaptic plasticity. In our study, the CaMKII inhibitor KN62 (3 μM), had no effect on NMDAR-LTD (63 ± 4%; n = 4; Figure 3F).

### Evidence that lipid kinases are not involved in LTD

We previously reported that activation of the lipid kinase PI3K is not required for LTD, based on the lack of sensitivity to wortmannin [19]. We have confirmed this finding using a different PI3K inhibitor, LY294002 (10 μM; 70 ± 3%; n = 5; Figure 3G). We also tested another kinase involved in lipid signalling, inositol 1,4,5-trisphosphate 3-kinase B (IP3K). The IP3K inhibitor was also without effect on LTD (20 μM; 64 ± 5%; n = 3; Figure 3H).

### Other protein kinases that are not involved in LTD

**Figure 4:**
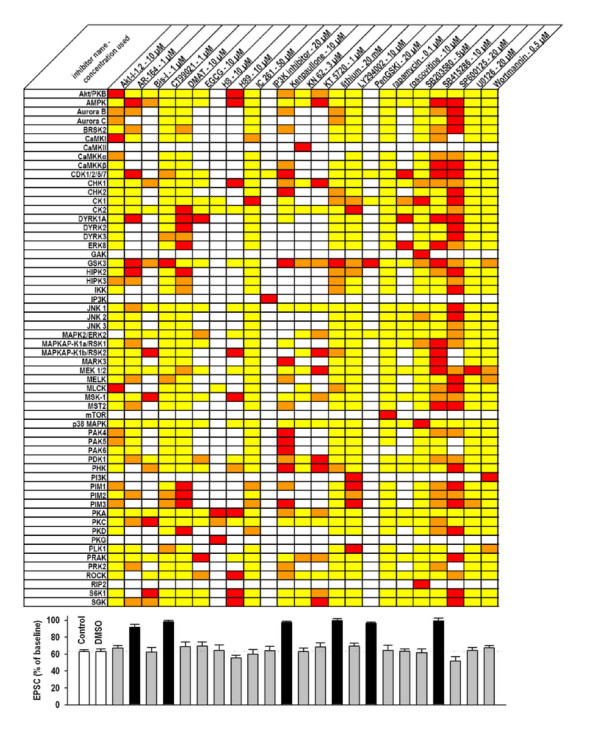
**Effect of inhibitors on various kinases and NMDAR-LTD**. The table shows the kinases (listed alphabetically) inhibited by each substance. The red cells depict a strong inhibition (over 66% inhibition), the orange a moderate inhibition (33–66%) and the yellow cells a weak inhibition (less than 33%). These data come from kinase assays performed at the same or a similar concentration as used in our experiments, except for AR-164 and KT5720 for which the kinase selectivity has been evaluated for a concentration 10-fold higher [24,54-58]. White cells indicate that specificity data are not available. The histogram shows the level of LTD, plotted as a percentage of baseline, for each of the conditions described immediately above. Black bars show a significant difference in LTD magnitude compared to control LTD (either with or without DMSO, as appropriate) and the gray bars show no difference with control.

No protein kinase inhibitor is entirely specific for one enzyme. In Figure 4 we present the selectivity information that is available for each of the inhibitors that we have used in this study and a previous one [19]. Data are also summarised in this Figure and the statistics are presented. Thus, by using a panel of 23 inhibitors, we have also shown that the activity of at least 57 kinases is not required for hippocampal NMDAR-LTD. Among these kinases, around 40 have not previously been studied in this respect: protein kinase AMP-activated (AMPK), Aurora kinase B, Aurora kinase C, BR serine/threonine kinase 2 (BRSK2), calcium/calmodulin-dependent protein kinase I (CaMKI), CaMK kinase (CaMKK) α and β, some cyclin dependent kinases (CDK), checkpoint kinase (CHK) 1 and 2, dual-specificity tyrosine-(Y)-phosphorylation regulated kinase (DYRK) 2 and 3, mitogen-activated protein kinase 15 (ERK8), cyclin G associated kinase (GAK), homeodomain interacting protein kinase (HIPK) 2 and 3, I-kappa B Kinase (IKK), mitogen-activated protein kinase 1 (MAPK2/ERK2), ribosomal protein S6 kinase, 90 kDa, polypeptide 1 and 3 (MAPKAP-K1a/RSK1 and MAPKAP-K1b/RSK2, respectively), MAP/microtubule affinity-regulating kinase 3 (MARK3), maternal embryonic leucine zipper kinase (MELK), myosin light chain kinase (MLCK), ribosomal protein S6 kinase, polypeptide 5 (MSK-1), serine/threonine kinase 3 (MST2), p21-activated kinase (PAK) 4, 5 and 6, 3-phosphoinositide dependent protein kinase-1 (PDK1), phosphorylase kinase (PHK), pim-1, pim-2 and pim-3 oncogene (PIM1, PIM2 and PIM3, respectively), protein kinase D (PKD), polo-like kinase 1 (PLK1), MAP kinase-activated protein kinase 5 (PRAK), protein kinase N2 (PRK2), Rho-associated coiled-coil containing protein kinase (ROCK), receptor-interacting serine-threonine kinase 2 (RIP2), ribosomal protein S6 kinase, 70 kDa (S6K1) and serum/glucocorticoid regulated kinase (SGK).

## Discussion

The primary conclusion of the present study, together with our previous work [19], is that of 58 ser/thr protein kinases investigated we found evidence for the involvement of only one, GSK-3 in LTD. Our studies focused on NMDAR-LTD at CA3-CA1 synapses of two-week-old rats, used a pairing protocol to induce LTD within single neurons and were performed at room temperature. Whilst this represents a fairly standard protocol, we cannot exclude a role of the other protein kinases in other neuronal pathways or at CA1 synapses under different experimental conditions.

To study a panel of inhibitors individually (in our case 23) via inclusion in the whole-cell solution is an extremely labour-intensive approach, which has not been applied previously in the study of synaptic plasticity. We believe, however, that such a strategy is vitally important due to the relative non-selectivity of most protein kinase inhibitors. For example, KT5720, a commonly used PKA inhibitor, is more potent on 7 other kinases, described in Figure 4, than it is on PKA.

### GSK-3

Our results confirm that GSK-3 plays an essential role in hippocampal LTD. In the present study we have used three of the most selective GSK-3 inhibitors that are available. Most GSK-3 inhibitors also inhibit the closely related cyclin-dependent kinases (CDKs). However, inhibition of CDKs cannot explain the block of LTD since, firstly, the GSK-3 inhibitor lithium does not affect CDKs yet blocks LTD [19] and, secondly, the pan-CDK inhibitor roscovitine has no effect on LTD [19]. Furthermore, AR-164 is over 100 fold more potent on GSK-3 (Ki = 9 nM) than CDK1 (Ki = 1.4 μM). In total we have now tested six structurally distinct inhibitors of GSK-3. Inspection of Figure 4 shows that the block of LTD is extremely unlikely to be due to off-target effects of these inhibitors

### Other CMGC group kinases and CKI

It has been suggested that NMDAR-LTD involves activation of p38 MAPK [27]. However, in agreement with other studies [28-30], we are of the view that p38 MAPK is important for mGluR-LTD [31,32] rather than NMDAR-LTD in the hippocampus. We also obtained no evidence for a role of either JNK or ERK in NMDAR-LTD; kinases that have also been implicated in mGluR-LTD in the hippocampus [33,34].

DYRK1A is of interest because it has been linked to Down's syndrome and is expressed in the developing and mature brain [35]. Transgenic mice expressing human DYRK1A show impairment in hippocampal-dependent memory and a modification of both LTP and LTD [36]. However, the lack of effect of four inhibitors able to affect DYRK1A, strongly suggest that this enzyme is not directly involved in NMDAR-LTD.

Previous work has suggested that CK2 is involved in the regulation of NMDAR-mediated synaptic transmission and LTP but not LTD [37]. Our findings confirm that CK2 is not involved in LTD. Additionally, we extend these results by showing that CK1 is also not involved in LTD, based on the lack of effect of three inhibitors that are able to potently inhibit this kinase.

### AGC group kinases

Whilst most evidence implicates PKA and PKC in LTP [26] there are also indications for roles in LTD. Indeed, LTD is absent in mice in which PKA subunits have been knocked out [15,16] and LTD is blocked in wildtype mice by treatment with KT5720 or H89 [15,38]. Conversely, other work has suggested that dephosphorylation of a PKA substrate, ser845 of GluA1, is involved in NMDAR-LTD [39]. This site is believed to be phosphorylated to maintain basal synaptic transmission, such that inhibition of PKA function can mimic and occlude LTD [39,40]. Our results, showing that PKA is not implicated in LTD, do not concord with either of these positions [41].

It has been proposed that PICK1, a protein that binds PKCα [42], is involved in NMDAR-LTD [13,43,44] but see [45]. Our finding that a PKC inhibitor failed to affect NMDAR-LTD is consistent with previous work [10,43,46] and suggests that any acute role of PICK1 in NMDAR-LTD is independent of PKC.

The PKG signalling pathway has been implicated in LFS-induced LTD in the dentate gyrus [47]. However, the authors showed that the LTD induced by activation of the cGMP/PKG pathway was dependent on mGluRs, rather than NMDARs. In agreement with this study, we show that PKG is not involved in NMDAR-LTD at CA1 synapses.

Akt (PKB) is a downstream effector of PI3K and an upstream regulator of GSK-3. Our previous work suggested that Akt was not involved in NMDAR-LTD *per se*, rather that it was part of a mechanism that enables cross-talk between NMDAR-LTP and NMDAR-LTD [19]. Consistent with no direct involvement in LTD, we found no effect of an Akt inhibitor on this process.

### CaMKII

Our observation that LTD was unaffected by an inhibitor of CaMKII is also consistent with another study that applied the inhibitor directly into the postsynaptic neuron [48]. In the latter study, it was found that LTD was inhibited by the bath application of KN-62, suggesting that LTD may require activation of CaMKII located presynaptically (see also [49]).

### Lipid kinases

In agreement with our previous work, we found that inhibitors of PI3K had no effect on NMDAR-LTD [10,19] rather they enabled a heterosynaptic form of LTD [10]. In the present study we also found no involvement of the related kinase IP3K, an enzyme that is enriched in hippocampal dendritic spines [50,51]. Interestingly, previous work suggested an involvement of IP3K in NMDAR-dependent plasticity and LTP [52,53] but whether IP3K is also involved in NMDAR-LTD was hitherto not known.

## Conclusion

By use of a panel of inhibitors we have been able to discount a role of at least 57 ser/thr protein kinases in NMDAR-LTD at CA1 synapses. We suspect that several of the kinases that have previously been implicated in this form of LTD, such as PKA, can be explained by off-target effects of the inhibitors used. Of course, a modulatory role of these kinases that is only seen under certain experimental conditions cannot be excluded. Our experiments do, however, strongly suggest that GSK-3 is required for this form of LTD.

## Competing interests

The authors declare that they have no competing interests.

## Authors' contributions

SP and CSN conducted the electrophysiology experiments. ZAB participated in the electrophysiology experiments. RVB participated in the production of the AR-164. WJR and AJH participated in the production of PenGSKi and PenCTRL. PD, SMF and GLC wrote the manuscript. GLC supervised the entire project. All authors read and approved the final manuscript.
